# Prevalence of Hepatitis E Virus in Swine Fed on Kitchen Residue

**DOI:** 10.1371/journal.pone.0033480

**Published:** 2012-03-23

**Authors:** Peng Xiao, Ruiwen Li, Ruiping She, Jun Yin, Wengui Li, Jingjing Mao, Quan Sun

**Affiliations:** 1 Department of Veterinary Pathology, Key Laboratory of Zoonosis of Ministry of Agriculture, College of Veterinary Medicine, China Agricultural University, Beijing, China; 2 College of Traditional Veterinary Medicine, Hebei Agricultural University, Dingzhou, China; 3 College of Animal Science, Yunnan Agricultural University, Kunminng, China; Institute for Animal Health, United Kingdom

## Abstract

The aim of this study was to investigate the prevalence of swine hepatitis E virus (HEV) in pigs fed different feedstuffs (kitchen residue or mixed feeds) and genetic identification of HEV isolated in Hebei province, China. Serum and fecal samples were collected from adult swine. Anti-HEV antibody was evaluated by double sandwich antigen enzyme immunoassay. HEV RNA was extracted from fecal samples and amplified by nested RT-PCR. The reaction products were sequenced, and the sequence analyzed. Virus-like particles were distinguishable by negative staining in the electron microscope. Histopathological observation and immunohistochemical localization were used in the animal models. Overall, the anti-HEV positive percentage of serum samples from pigs fed on kitchen residue was 87.10% (27/31), and 53.06% (130/245) from pigs fed on complete feed. The HEV RNA positivity rate of fecal samples from pigs fed on kitchen residue was 61.54% (8/13), but zero for pigs fed on complete feed. Sequence analysis of these eight samples and comparison with the published sequence showed that there were eight groups that belonged to genotype 4 d and the nucleotide identity was 95.6–99.3%. swHE11 is most closely related to strain CCC220, and the other seven HEV isolates were most closely related to strains swGX40, SwCH189 and V0008ORF3, which are isolates from human and pigs. Histopathological observation showed that there was liver damage in the experimental group, and immunohistochemistry indicated that the HEV antigens were strongly positive at 7 days after infection. The results demonstrated that the prevalence of HEV in pigs fed on kitchen residue was higher than in those fed on complete feed (P<0.05).

## Introduction

Hepatitis E virus (HEV), the causative agent of hepatitis E, has a single-stranded, positive-sense RNA genome within a non-enveloped capsid [1]. HEV is classified as the sole member of the genus Hepevirus in the family Hepeviridae. The genome is approximately 7.2 kb in size and consists of a short 5′-untranslated region (UTR), three partially overlapping open reading frames (ORF1–3) and a short 3′-UTR terminated by a poly (A) tract [2]. ORF1 encodes for non-structural proteins such as methyltransferase, helicase and RNA-dependent RNA polymerase; ORF2 encodes the capsid protein; and ORF3 encodes the cytoskeleton-associated phosphoprotein [3], [4].

Hepatitis E is endemic in human populations in many developing regions of the world including Asia, Africa and Latin America [5]–[12]. In those regions, the disease is a serious public health problem. Most often, the infection appears as acute hepatitis with jaundice but with low mortality; however, in pregnant women, HEV can lead to fulminant acute hepatic failure [13]. In developed countries, HEV infection is sporadic in humans but has become increasingly important [14]–[19].

Accumulating evidence indicates that there are animal reservoirs of HEV and that hepatitis E is a zoonotic disease [20]. The Meng isolate was the first reported strain from an animal, namely, an infected pig in the United States in 1997 [21]. Since then, many swine HEV isolates have been identified in numerous countries [20], [22]–[29]. In addition, there have been reports of high genetic relatedness between HEV isolates obtained from humans and those from swine in the same geographical regions [30].

Hepatitis E is endemic in swine herds in China, and in suburban areas there is a history of feeding pigs with kitchen residue from restaurants. However, epidemiological data of HEV prevalence in swine herds is insufficient in Hebei province, China, and the prevalence of HEV in pigs fed with kitchen residue is not clear.

In the current study, we investigated HEV infection among swine fed with kitchen residue by ELISA and nested RT-PCR in Hebei province, and compared it with HEV prevalence in pigs fed with complete feed.

## Materials and Methods

### Sample Collection

A total of 245 serum samples were collected from adult swine fed with complete feed, including 113 from two swine farms in Shijiazhuang and 132 from Baoding. In addition, 31 serum samples were collected from pigs fed with kitchen residue. All the above samples were from Hebei province, China in August 2008. Serum samples were stored at −80°C until testing for HEV antibodies.

Fifty-six fecal samples were collected from younger pigs (2 or 3 months old) from Baoding, Hebei in November 2008, including 13 from an individual herd fed with swill, and 43 individual samples from a herd fed with feeder. Suspensions of fecal samples were prepared by vortexing 1 g of feces with 10 ml PBS. The supernatants were stored at −80°C pending use.

### ELISAs for Detecting Anti-HEV Antibodies

All the serum samples were evaluated for anti-HEV antibodies using a commercial ELISA diagnostic kit (Wantai Biological Pharmacy Co., Beijing, China). All assay procedures were carried out following the manufacturer's instructions. The samples with optical density (OD) less than the cutoff value (mean OD for the three negative controls on each plate plus 0.12) were considered negative. Samples with OD greater than or equal to the cutoff value were tentatively considered positive and then retested to confirm the result. The absorbance was determined at 450/620 nm (Multiscan Titertek MCC; Eflab Oy, Finland).

### Genetic and Phylogenetic Analysis

RNA extraction and reverse transcription was carried out as described previously [31]. Viral RNA was extracted from the samples with the TRIZOL LS kit (Invitrogen, Carlsbad, CA, USA) according to the manufacturer's instructions. Nested RT-PCR was performed using ORF2-specific primers (Table 1). PCR products were purified and cloned into the pMD18-T vector (Takara Dalian Co. Ltd., China), and cDNA templates were sequenced on an ABI377 automated DNA sequencer using reagents from BGI Life Tech Co. Ltd. (Beijing, China). The nucleotide sequences were compiled, edited and compared by using the DNAMAN program (version 6.0; Lynnon BioSoft, USA). Phylogenetic analysis was carried out by the neighbor-joining bootstrap analysis (1000 replicates) using the MEGA program (version 4.1; http://www.megasoftware.net). The nucleotide sequences from this study are available from GenBank under the accession numbers listed in Table S1.

**Table 1:** Primers used for nested RT-PCR.

	primer	Tm	Product size
HE164F1	5′-GCR GTG GTT TCT GGG GTG AC-3′	58°C	164 bp
HE164R1	5′-CTG GGM YTG GTC DCG CCA AG-3′		
HE137F2	5′-GYT GAT TCT CAG CCC TTC GC-3′		137 bp
HE137R2	5′-GMY TGG TCD CGC CAA GHG GA-3′		

### Immune Electron Microscopy

To obtain ultrastructural evidence for the presence of HEV-related viral particles in swine feces containing ORF2 antigen, HEV ORF2-antigen-positive samples were collected and viral particles in feces of infected swine were morphologically analyzed by electron microscopy. Feces negative for HEV ORF2 antigen served as controls. Diluted virus-positive stool samples were centrifuged at 4000 rpm for 30 min, then 0.01 M polyethylene glycol 6000 was added to the subsequent upper aqueous phase. After Supernatant added an appropriate dilution of anti-HEV antibodies overnight at 4°C, the samples were centrifuged at 20,000 rpm for 1 hour, re-suspended in PBS and stained for 1 minute with 1% uranyl acetate. All electron micrographs were obtained with a JEV1230 transmission electron microscope (JEOL Ltd., Tokyo, Japan) at 80 kV.

### Animals and Experimental Protocols

To assess the pathogenicity of HEV in animals, 8-week-old male Mongolian gerbils, purchased from Experimental Animal Center, Capital Medical University, Beijing, China (certificate number 27389), were housed in microisolator cages ventilated under negative pressure with HEPA-filtered air. During the experiment, gerbils had access to food and water *ad libitum*. The gerbils were inoculated intraperitoneally with HEV-positive serum. Mock-infected control animals were inoculated intraperitoneally with an equivalent dilution of normal saline NS. At 1, 2, 4 and 8 weeks after inoculation, five gerbils were sacrificed in each group, and liver tissue samples were collected and fixed for histopathological observation and immunohistochemical localization. All experimental procedures were approved by the Institutional Animal Care and Committee of China Agricultural University (The certificate of Beijing Laboratory Animal employee, ID: 20070371).

### Statistical Analysis

Descriptive statistical analysis was performed using SPSS for Windows 13.0 (SPSS Inc., Chicago, IL, USA), and the χ^2^ test was used to analyze the prevalence difference between the breeding environments.

## Results

### Prevalence of IgG Anti-HEV in Swine Serum in Hebei Province

**Figure 1:**
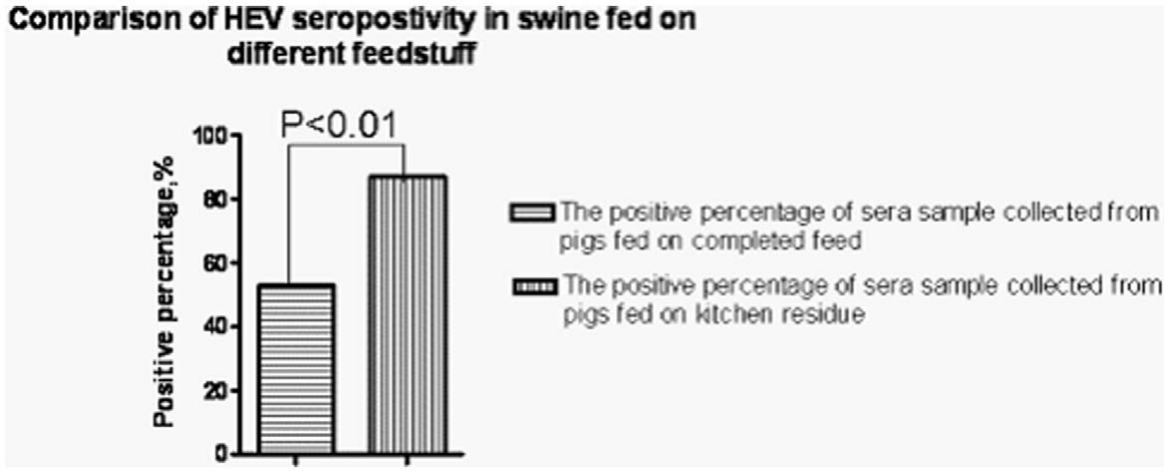
Comparison of HEV seropositivity in swine fed on different feedstuff. The percentage of positive serum samples from pigs fed on kitchen residue was clearly higher than that from those fed on complete feed.

The overall prevalence of anti-HEV positive antibodies in swine sera was 56.88% (157/276) across the regions of Hebei province. Anti-HEV antibody prevalence ranged from 42.86% to 87.10%; the distribution of prevalence is shown in Figure 1. Anti-HEV antibodies were detected in 87.10% (27/31) of pigs fed with swill, but the rate was only 53.06% (130/245) in pigs fed with feeder. The percentage of positive serum samples from pigs fed with swill was clearly greater than that from pigs fed with feeder (Table 2).

**Table 2:** Detection of HEV antibodies in swine sera in Hebei province, China.

	Farm1	Farm2	Farm3	Farm4	Farm5	Total
Sera number	89	24	104	28	31	276
Positive number	46	13	59	12	27	157
Positive percentage	51.59%	54.17	56.73%	42.86%	87.10%	56.88%

Note: Farm1, farm2, farm3 and farm4 are large farms in which pigs were fed with feeder; Farm1 and farm2 were in Shijianzhuang province, and farm3 and farm4 in Baoding province; farm5 was a family-size farm in which pigs were fed with swill.

### Nested RT-PCR Detection and Phylogenetic Analysis

**Figure 2:**
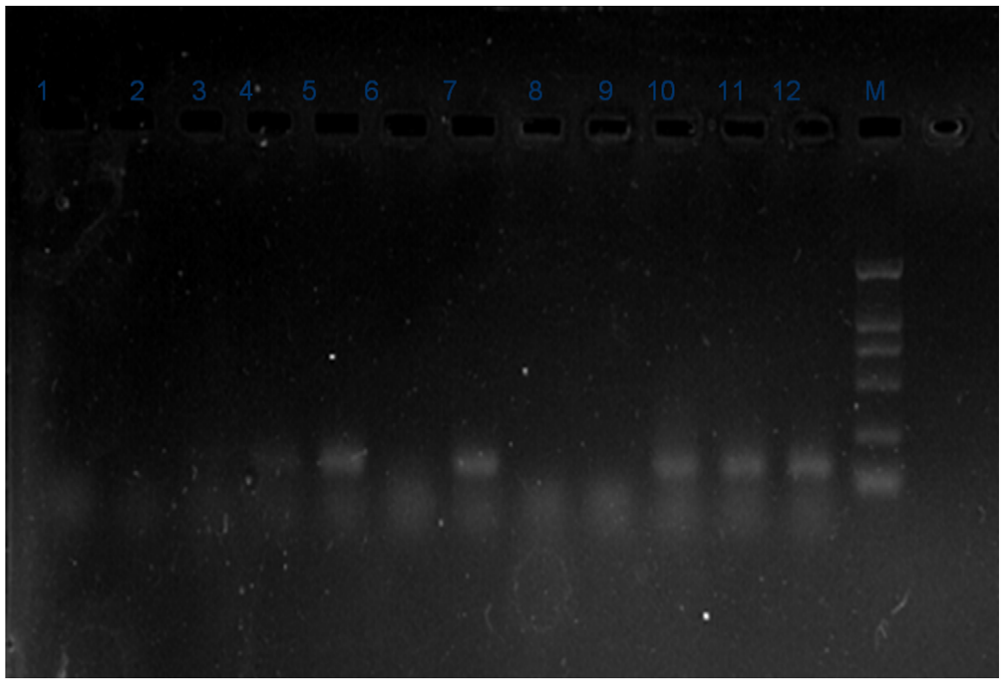
HEV RNA detection by nested RT-PCR. Results for fecal samples.

Eight of the 56 fecal samples (14.29%) were positive for HEV RNA. All eight positive samples were from the 13 collected from pigs fed with swill, and none of the 43 fecal samples collected from pigs fed with feeder were positive for HEV RNA (Figure 2). HEV RNA sequences were designated as swHB3–5, 7, 8 and 11–13, and deposited in DDBJ/EMBL/GenBank (accession nos. GU553094–GU553101, Table 3). Eight HEV isolates, swHE3–5, 7, 8 and 11–13, had the highest identity with strains isolated from humans (ccc220, AB108537; V0080RF3, AB075971; 94.9–99.3% identity). The identity within the eight HEV isolated sequences was 95.6–99.3%. The eight HEV isolated nucleotide sequences shared 81.8–83.2%, 47.6–50.0%, 82.5–86.1% and 94.9–97.8% identity with strains representing genotype 1 (HEBEI, M94177), 2 (M1, M74506), 3 (HEV-US1, AF060668) and 4 (CCC220, AB108537), respectively. The mean genetic distances between the eight HEV isolates and four HEV genotypes were 0.119, 0.403, 0116 and 0.048, respectively.

**Table 3:** Background information of swine HEV examined in this study.

Virus	Abbreviation	Date of isolation	GenBank accession No.
IV/Swine/Hebei/3/10	swHB-3	May 2010	GU553094
IV/Swine/Hebei/4/10	swHB-4	May 2010	GU553095
IV/Swine/Hebei/5/10	swHB-5	May 2010	GU553096
IV/Swine/Hebei/7/10	swHB-7	May 2010	GU553097
IV/Swine/Hebei/8/10	swHB-8	May 2010	GU553098
IV/Swine/Hebei/11/10	swHB-11	May 2010	GU553099
IV/Swine/Hebei/12/10	swHB-12	May 2010	GU553100
IV/Swine/Hebei/13/10	swHB-13	May 2010	GU553101

To determine the genetic and evolutionary characterization of HEV isolated from pigs in Hebei province, all eight gene segments from each of eight isolates were sequenced and phylogenetically analyzed. The nucleotide sequences were compared with the sequences of other representative viruses of swine and human HEVs obtained from GenBank.

**Figure 3:**
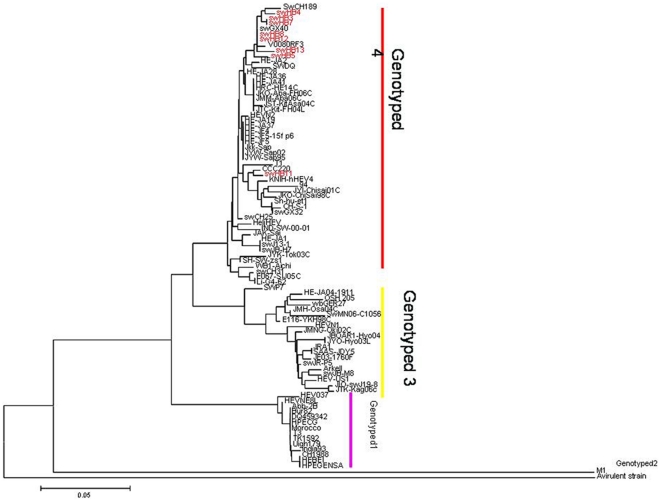
Phylogenetic tree of ORF2/ORF3 of HEV isolated from swine fed on kitchen residue. Swine HEV isolated as part of this study are shown in capitals and are labeled SWHB-3–5, 7, 8 and 11–13. The scale bar represents an evolutionary distance of 0.05 nucleotides per position.

As reported in previous genetic studies, HEV mainly comprises four genotypes. These viruses include genotype I represented by HEBEI, M94177; genotype II represented by M1, M74506; genotype III represented by HEV-US1, AF060668; and genotype IV represented by CCC220, AB108537. Since the first isolation of swine HEV from the Mid-West United States in 1997, these viruses have evolved gradually and conservatively. HEV genotype IV has been reported to be prevalent in China in recent years. In the present study, phylogenetic analysis of genes showed that all eight isolates shared 95.6–99.3% identity with each other, and all belonged to the genotype IV lineage. swHE11 was most closely related to strain CCC220, and the other seven HEV isolates were most closely related to swGX40, swCH189 and V0008ORF3, which have been isolated from humans and pigs (Figure 3).

### Negative Staining of Samples Observed by Electron Microscopy

In positive fecal samples from swine fed on kitchen residue, electron microscopy revealed clusters of virus-like particles, 27–34 nm in diameter, whose shape was consistent with that described in previous studies (figure not shown).

### Histopathological Observation

**Figure 4:**
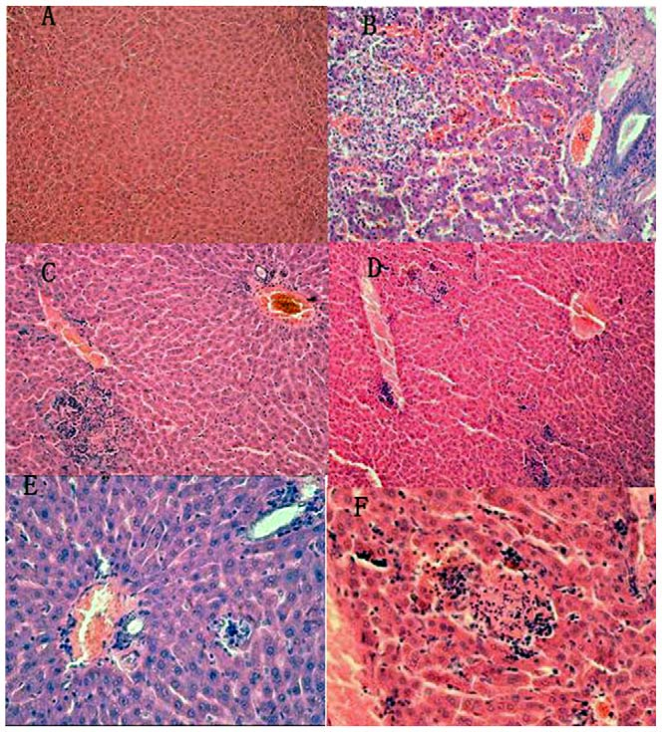
Histopathological analysis of liver sections stained with hematoxylin and eosin (HE). A: liver structure is clear and the liver cells are in order (control group; HE, 20×). B: Sinus congestion, proliferation of fibrous connective tissue in portal area (14 days post-infection; HE 40×). C: Hepatocyte necrosis and lymphocyte infiltration (7 days post-infection; HE, 40×). D: Multiple lymphocyte infiltration (7 days post-infection; HE, 10×). E: Lymphocytic infiltration in Kiernan's space (28 days post-infection; HE, 20×). F: Lobular necrotic nodules (28 days post-infection; HE, 40×).

Histopathological observation showed that the liver from gerbils in the control group had no significant disease; the structure was clear and the liver cells were in order. In the experimental groups, hepatic lesions were observed at 7 days, with lymphocytic infiltration in Kiernan's space, and degeneration of hepatocyte granules. Focal lymphocytic invasion appeared at 14 days after inoculation, and liver cell cord disorder and bile duct proliferation. After 28–64 days, there were signs of multiple lymphocyte infiltration, hepatocyte necrosis, increased Kupffer cells, and proliferation of fibrous connective tissue (Figure 4).

### Immunohistochemistry

**Figure 5:**
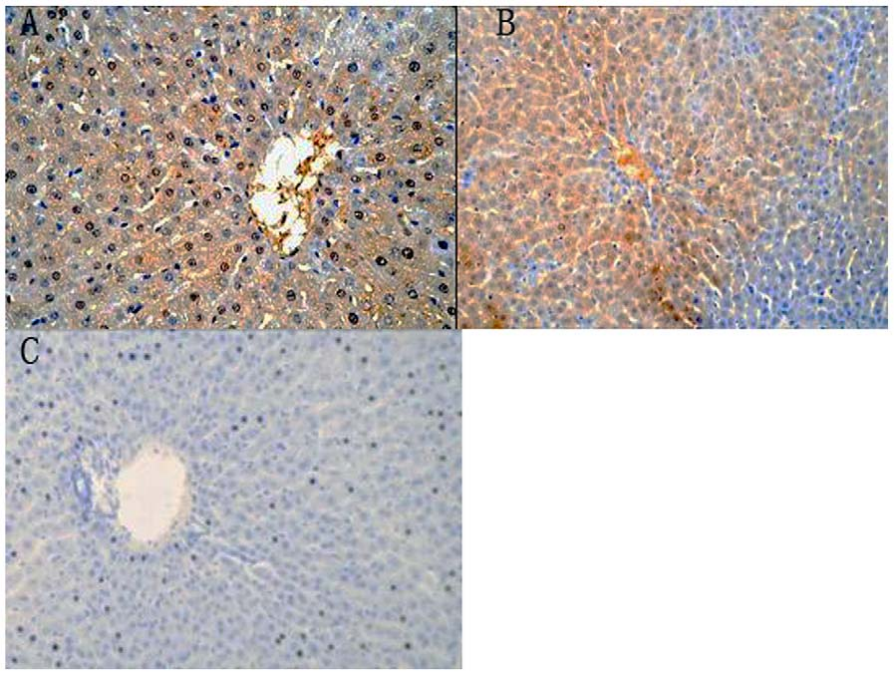
Immunohistochemical staining of HEV proteins in the liver. A: Strong positive signals in the cytoplasm (40×). B: Sheets of liver cells were positive for HEV proteins (20×). C: No positive signal (control group; 20×).

Immunohistochemistry showed strong positive staining for HEV antigen in the liver of gerbils at 7 days after infection. Granular or diffuse positive staining was seen in the cytoplasm and nucleus of liver cells. HEV nuclear antigen positive exists in binuclear hepatocyte. Those liver cells were positive for HEV antigen and were often larger than normal. HEV-positive granules were seen in Kupffer cells and biliary epithelial cells in the portal area, but no positive cells were observed in the control group (Figure 5).

## Discussion

Since the first swine strain was characterized from a pig in the United States, HEV infection in pigs appears to be widespread throughout the world. Although direct evidence of HEV transmission from animals to humans is lacking, transmission of HEV from humans to swine has been proven experimentally [31]. Furthermore, rhesus monkeys have been found to support replication of human HEV genotypes 1, 2 and 3, and chimpanzees can be infected with the swine HEV strain (swUS) [32]. This raises the possibility of swine HEV infecting humans, especially in regions where highly conserved sequences have been isolated from both humans and swine. Moreover, sporadic human cases of acute HEV infection linked with consumption of raw or insufficiently cooked meat (boar or deer) have been reported in Japan [33]. In cases of acute hepatitis E contracted by consumption of infected meat, 100% identity between isolates from humans and infected animals has been observed [34]. In France, isolation of a virus with related HEV sequences from a patient and his pet pig suggests that the most likely route of transmission was from pig to human [35]. Therefore, pigs should be considered a reservoir for HEV and exposure to feces from infected pigs represents a risk for transmission of HEV to other pigs and possibly to other species, including humans. The evidence strongly suggests that swine are a common source of human HEV infection. However, the source of HEV infection for such animals is not known and is still being investigated.

Our previous immunohistochemical study showed that HEV antigen was detected more frequently in slaughtered swine from suburban rather than remote areas [36]. That study also showed significantly higher anti-HEV prevalence in family-size farms compared with large farms (76.6% vs. 90%) [36]. The present study showed that the HEV prevalence in pigs fed with kitchen residue was greater than in those fed with feeder. The seropositivity of HEV IgG was 56.88% (157/276) in swine in some districts of Hebei province. The positive rate for anti-HEV antibodies was 87.10% (27/31) in pigs fed with swill, but it was 53.06% (130/245) in those fed with feeder. The detection rate of HEV RNA in swine feces was 61.54% (8/13) in pigs fed with swill, but it was not detected in those fed with feeder,and is higher than the pre-anthors. We took fecal samples from both the large piggery and farmers' backyards. The large piggery is geographically near the farmers' backyards that are used for feeding pigs, mainly on swill. Both of the culture environment and the pig species are almost the same. However, the feeding sources were different: the large piggery used compound feed and the farmers fed their pigs on swill from a school. The genotype analysis also demonstrated that all the eight strains (swHB3–5, 7, 8 and 11–13)belonged to genotype IV. The amino acid sequence of these strains had more than 96.1–96.4% identity compared to human strains, such as between strains swHE11 and CCC220. The food waste from the school canteens was only slightly heated and was not completely sterile. This may be the main reason why HEV prevalence was higher in pigs fed on kitchen residue compared with conventional pig feed, but the specific pathway and mechanism of infection remain elusive and need further study.

We detected the presence of 27–34-nm diameter virus-like particles in HEV RNA-positive fecal samples, using immune electron microscopy, which is consistent with previous studies [37]. Histological studies and immunohistochemical staining revealed hepatic lesions in gerbils experimentally infected with HEV, which were similar to infection in pigs and humans.

Further investigation is required in additional herds fed on kitchen residue to determine the prevalence of swine HEV, the genotypic relationship to other Chinese strains, and the relations with human strains of HEV. In particular, we examined pigs that were fed on swill, and our results demonstrated that these pigs were more likely to be infected with HEV. In addition, more attention should be given to the safety of pork products made from pigs that are fed on swill.

## Supporting Information

Table S1 HEV strains used in the phylogenetic and sequence analyses.
(DOC)

## References

[1] Emerson SU, Nguyen H, Graff J, Stephany DA, Brockington A (2004). In vitro replication of hepatitis E virus (HEV) genomes and of an HEV replicon expressing green fluorescent protein.. J Virol.

[2] Tam AW, Smith MM, Guerra ME, Huang CC, Bradley DW (1991). Hepatitis E virus (HEV): molecular cloning and sequencing of the full-length viral genome.. Virology.

[3] Jameel S, Zafrullah M, Ozdener MH, Panda SK (1996). Expression in animal cells and characterization of the hepatitis E virus structural proteins.. J Virol.

[4] Zafrullah M, Ozdener MH, Panda SK, Jameel S (1997). The ORF3 protein of hepatitis E virus is a phosphoprotein that associates with the cytoskeleton.. J Virol.

[5] Aye TT, Uchida T, Ma XZ, Iida F, Shikata T (1992). Complete nucleotide sequence of a hepatitis E virus isolated from the Xinjiang epidemic (1986–1988) of China.. Nucleic Acids Res.

[6] Huang RT, Li DR, Wei J, Huang XR, Yuan XT (1992). Isolation and identification of hepatitis E virus in Xinjiang, China.. J Gen Virol.

[7] Hyams KC, McCarthy MC, Kaur M, Purdy MA, Bradley DW (1992). Acute sporadic hepatitis E in children living in Cairo, Egypt.. J Med Virol.

[8] Zhang GQ, Tian GS (1993). A preliminary report on the prevalence of different types of hepatitis in adult patients with acute sporadic hepatitis in Beijing area.. Zhonghua Nei Ke Za Zhi.

[9] Corwin AL, Khiem HB, Clayson ET, Pham KS, Vo TT (1996). A waterborne outbreak of hepatitis E virus transmission in southwestern Vietnam.. Am J Trop Med Hyg.

[10] Emerson SU, Purcell RH (2004). Running like water–the omnipresence of hepatitis E.. N Engl J Med.

[11] Shukla P, Chauhan UK, Naik S, Anderson D, Aggarwal R (2007). Hepatitis E virus infection among animals in northern India: an unlikely source of human disease.. J Viral Hepat.

[12] Purcell RH, Emerson SU (2000). Hepatitis E virus infection.. Lancet.

[13] Kumar RM, Uduman S, Rana S, Kochiyil JK, Usmani A (2001). Sero-prevalence and mother-to-infant transmission of hepatitis E virus among pregnant women in the United Arab Emirates.. Eur J Obstet Gynecol Reprod Biol.

[14] Mizuo H, Suzuki K, Takikawa Y, Sugai Y, Tokita H (2002). Polyphyletic strains of hepatitis E virus are responsible for sporadic cases of acute hepatitis in Japan.. J Clin Microbiol.

[15] Mast EE, Kuramoto IK, Favorov MO, Schoening VR, Burkholder BT (1997). Prevalence of and risk factors for antibody to hepatitis E virus seroreactivity among blood donors in Northern California.. J Infect Dis.

[16] Mateos ML, Teruel JL, Sierra MP, Gazapo E (1997). High prevalence of hepatitis E virus antibodies in Spanish hemodialysis patients.. Nephron.

[17] Mansuy JM, Legrand-Abravanel F, Calot JP, Peron JM, Alric L (2008). High prevalence of anti-hepatitis E virus antibodies in blood donors from South West France.. J Med Virol.

[18] Dalton HR, Bendall R, Ijaz S, Banks M (2008). Hepatitis E: an emerging infection in developed countries.. Lancet Infect Dis.

[19] Dalton HR, Fellows HJ, Gane EJ, Wong P, Gerred S (2007). Hepatitis E in new zealand.. J Gastroenterol Hepatol.

[20] Lu L, Li C, Hagedorn CH (2006). Phylogenetic analysis of global hepatitis E virus sequences: genetic diversity, subtypes and zoonosis.. Rev Med Virol.

[21] Meng J, Dubreuil P, Pillot J (1997). A new PCR-based seroneutralization assay in cell culture for diagnosis of hepatitis E.. J Clin Microbiol.

[22] Feagins AR, Opriessnig T, Guenette DK, Halbur PG, Meng XJ (2007). Detection and characterization of infectious Hepatitis E virus from commercial pig livers sold in local grocery stores in the USA.. J Gen Virol.

[23] Bouwknegt M, Lodder-Verschoor F, van der Poel WH, Rutjes SA, de Roda Husman AM (2007). Hepatitis E virus RNA in commercial porcine livers in The Netherlands.. J Food Prot.

[24] Caprioli A, Martelli F, Ostanello F, Di Bartolo I, Ruggeri FM (2007). Detection of hepatitis E virus in Italian pig herds.. Vet Rec.

[25] Cheng PN, Wang RH, Wu IC, Wu JC, Tseng KC (2007). Seroprevalence of hepatitis E virus infection among institutionalized psychiatric patients in Taiwan.. J Clin Virol.

[26] Lorenzo FR, Tsatsralt-Od B, Ganbat S, Takahashi M, Okamoto H (2007). Analysis of the full-length genome of hepatitis E virus isolates obtained from farm pigs in Mongolia.. J Med Virol.

[27] Chobe LP, Lole KS, Arankalle VA (2006). Full genome sequence and analysis of Indian swine hepatitis E virus isolate of genotype 4.. Vet Microbiol.

[28] Munne MS, Vladimirsky S, Otegui L, Castro R, Brajterman L (2006). Identification of the first strain of swine hepatitis E virus in South America and prevalence of anti-HEV antibodies in swine in Argentina.. J Med Virol.

[29] Ning H, Niu Z, Yu R, Zhang P, Dong S (2007). Identification of genotype 3 hepatitis E virus in fecal samples from a pig farm located in a Shanghai suburb.. Vet Microbiol.

[30] Inoue J, Takahashi M, Yazaki Y, Tsuda F, Okamoto H (2006). Development and validation of an improved RT-PCR assay with nested universal primers for detection of hepatitis E virus strains with significant sequence divergence.. J Virol Methods.

[31] Williams TP, Kasorndorkbua C, Halbur PG, Haqshenas G, Meng XJ (2001). Evidence of extrahepatic sites of replication of the hepatitis E virus in a swine model.. J Clin Microbiol.

[32] Meng XJ, Halbur PG, Shapiro MS, Govindarajan S, Bruna JD (1998). Genetic and experimental evidence for cross-species infection by swine hepatitis E virus.. J Virol.

[33] Tei S, Kitajima N, Ohara S, Inoue Y, Miki M (2004). Consumption of uncooked deer meat as a risk factor for hepatitis E virus infection: an age- and sex-matched case-control study.. J Med Virol.

[34] Yazaki Y, Mizuo H, Takahashi M, Nishizawa T, Sasaki N (2003). Sporadic acute or fulminant hepatitis E in Hokkaido, Japan, may be food-borne, as suggested by the presence of hepatitis E virus in pig liver as food.. J Gen Virol.

[35] Renou C, Cadranel JF, Bourliere M, Halfon P, Ouzan D (2007). Possible zoonotic transmission of hepatitis E from pet pig to its owner.. Emerg Infect Dis.

[36] Li W, She R, Wei H, Zhao J, Wang Y (2009). Prevalence of hepatitis E virus in swine under different breeding environment and abattoir in Beijing, China.. Vet Microbiol.

[37] Balayan MS, Andjaparidze AG, SS S (1983). Evidence for a virus in non-A, non-B hepatitis transmitted via the fecal-oral route. Intervirology 20(1):23–31.[J].. Proceedings of the National Academy of Sciences.

