# Supplementary material for: Prevalence of Hepatitis E Virus in Swine Fed on Kitchen Residue
Source: PLoS One. 2012 Mar 23;7(3):e33480. doi: 10.1371/journal.pone.0033480 (PMC3311637; doi:10.1371/journal.pone.0033480)
Supplement: Table S1 — HEV strains used in the phylogenetic and sequence analyses. (DOC) [file pone.0033480.s001.doc]

**Table S1.** HEV strains used in the phylogenetic and sequence analyses

| Type | Isolate | Country | Host | Accession number |
| --- | --- | --- | --- | --- |
| 1 | HEV037 | Indian | Human | X98292 |
|  | HEVNE8L | Burma | Human | D10330 |
|  | Abb-2B | Pakistan | Human | AF185822 |
|  | Bur82 | Burma | Human | M73218 |
|  | DQ459342 | Indian | Human | DQ459342 |
|  | HPECG | China | Human | D11092 |
|  | Morocco | Morocco | Human | AY230202 |
|  | T3 | Chad | Human | AY204877 |
|  | TK1592 | Nepal | Human | AF051830 |
|  | Uigh179 | China | Human | D11093 |
|  | India93 | India | Human | X99441 |
|  | CH1988 | China | Human | L08816 |
|  | HEBEI | China | Human | M94177 |
|  | HPEGENSA | China | Human | LO8816 |
| 2 | M1 | Mexico | Human | M74506 |
| 3 | SWP7 | Spain | Swine | EU723515 |
|  | HE-JA04-1911 | Japan | Human | AB248520 |
|  | Osh 205 | Kyrgyzstan | Swine | AF455784 |
|  | wbGER27 | Germany | Wild boar | FJ748531 |
|  | JMH-Osa04C | Japan | Human | AB291961 |
|  | SwMN06-C1056 | Mongolia | Swine | AB290313 |
|  | E116-YKH98C | Japan | Human | AB369687 |
|  | HEVN1 | Japan | Human | AB246676 |
|  | JMNG-Oki02C | Japan | Wild mongoose | AB236320 |
|  | JBOAR1-Hyo04 | Japan | Wild boar | AB189070 |
|  | JYO-Hyo03L | Japan | Human | AB189075 |
|  | JRA1 | Japan | Human | AP003430 |
|  | SAAS-JDY5 | China | Swine | FJ527832 |
|  | JE03-1760F | Japan | Human | AB437318 |
|  | swJR-P5 | Japan | Swine | AB481229 |
|  | Arkell | Canada | Swine | AY115488 |
|  | swJB-M8 | Japan | Swine | AB481228 |
|  | HEV-US1 | US | Swine | AF060668 |
|  | JIO-swJ19-8 | Japan | Swine | AB443627 |
|  | JTK-Kag06c | Japan | Human | AB291960 |
| 4 | swCH189 | China | Swine | FJ610232 |
|  | swGX40 | China | Swine | EU676172 |
|  | V0080RF3 | Vietnam | Swine | AB075971 |
|  | HE-JA2 | Japan | Human | AB220974 |
|  | swDQ | China | Swine | DQ279091 |
|  | HE-JA28 | Japan | Human | AB220976 |
|  | HE-JA36 | Japan | Human | AB220977 |
|  | HE-JA41 | Japan | Human | AB220979 |
|  | HRC-HE14C | Japan | Human | AB291965 |
|  | JKO-Aba-FH06C | Japan | Human | AB291967 |
|  | JMM-Aba06C | Japan | Human | AB2921968 |
|  | JST-KitAsa04C | Japan | Human | AB291966 |
|  | JTC-Kit-FH04L | Japan | Human | AB291959 |
|  | HEVN2 | Japan | Human | AB253420 |
|  | HE-JA37 | Japan | Human | AB220978 |
|  | HE-JA19 | Japan | Human | AB220975 |
|  | HE-JF4 | Japan | Human | AB220972 |
|  | HE-JF5-15f p6 | Japan | Culture supernatant | AB480825 |
|  | HE-JF5 | Japan | Human | AB220973 |
|  | Jkk-Sap | Japan | Human | AB074917 |
|  | JYW-Sap02 | Japan | Human | AB161719 |
|  | JYW-Sap95 | Japan | Human | AB161717 |
|  | HE-JF4 | Japan | Human | AB220972 |
|  | T1 | China Beijing | Human | AJ272108 |
|  | CCC220 | China | Human | AB108537 |
|  | KNIH-hHEV4 | Korea | Human | FJ763142 |
|  | 94 | China | Human | DQ068211 |
|  | JYI-Chisai01C | China | Human | AB197674 |
|  | JKO-ChiSai98C | China | Human | AB197673 |
|  | Sh-hu-et1 | China | Human | FJ373295 |
|  | CH-S-1 | China | Swine | EF077630 |
|  | swGX32 | China | Swine | EU366959 |
|  | swCH25 | China | Swine | AY594199 |
|  | HeljHEV | China | Swine | DQ294630 |
|  | IND-SW-00-01 | India | Swine | AY723745 |
|  | JAK-Sai | Japan | Human | AB074915 |
|  | HE-JA1 | Japan | Human | AB097811 |
|  | swJ13-1 | Japan | Swine | AB097811 |
|  | JYK-Tok03C | Japan | Human | AB291964 |
|  | SH-SW-zs1 | China | Swine | EF570133 |
|  | WB1-Aichi | Japan | Wild boar | DQ079628 |
|  | swCH31 | China | Swine | DQ450072 |
|  | Li-G4-62 | China | Human | DQ079631 |
|  | E067-SIJ05C | Japan | Human | AB369690 |
|  | Avirulent strain | US | Avian | EF206691 |
